# The utility of the 24-h delayed film of barium enema for detecting the dysganglionic bowel segment in Hirschsprung’s disease

**DOI:** 10.3389/fped.2022.979149

**Published:** 2022-09-20

**Authors:** Bingyan Zhou, Di Wang, Ke Chen, Yonghua Niu, Chunlei Jiao, Tianqi Zhu, Jiexiong Feng

**Affiliations:** ^1^Department of Pediatric Surgery, Tongji Hospital, Tongji Medical College, Huazhong University of Science and Technology, Wuhan, China; ^2^Hubei Clinical Center of Hirschsprung’s Disease and Allied Disorders, Wuhan, China

**Keywords:** Hirschsprung’s disease, 24-h delayed film of barium enema, rectal biopsy, resection strategy, dysganglionic bowel segment

## Abstract

**Background:**

Preoperative evaluation of the dysganglionic bowel segment is critical for establishing the optimal resection strategy for Hirschsprung’s disease (HSCR), which facilitates patient outcomes.

**Objective:**

We set out to determine the utility of the 24-h delayed film of barium retention in predicting the length of dysganglionic bowel segment in HSCR.

**Materials and methods:**

A retrospective study of patients with clinically suspicious HSCR who underwent a preoperative 24-h delayed film of barium enema and were surgically treated from January 2015 to December 2019 was conducted.

**Results:**

Two hundred and 58 patients were enrolled in this study. The sensitivity, specificity, positive and negative predictive values (NPVs) of the 24-h delayed film of barium enema to predict the neuropathological segment were 89.1, 91.5, 91.3, and 89.4%, respectively. The Youden index was 80.6%, with a kappa value of 0.806 (*P* < 0.001). The correlation rate between barium retention level and pathological results was 72.7% (16/22) when aganglionosis was restricted within the mid-distal rectum (short-segment type), increasing to 92.0% (46/50) and 93.5% (174/186) for patients that had aganglionosis extended beyond the mid-distal rectum (classical type) and sigmoid colon (long-segment type), respectively. Lastly, patients younger than 3 months showed a lower correlation rate (72.2%) compared to patients aged 3–12 months (91.0%) and > 12 months (92.6%).

**Conclusions:**

Our investigation of the 24-h delayed film of barium enema performed for patients suspected of having HSCR indicated that the barium retention level remains crucial in predicting dysganglionic bowel segment, which contributes to the decision-making for surgical physicians.

## Introduction

Hirschsprung’s disease (HSCR) is characterized by an absence of ganglion cells in the distal bowel due to the failure of craniocaudal migration of enteric neural crest cells during embryological development (1). It occurs about 1 in 5,000 live births (2). The treatment is primarily surgical resection of the inert terminal bowel by a pull-through operation (3). Nevertheless, it is increasingly recognized that many patients continue to suffer from the complications that occurred following the surgical repair, especially the obstructive symptoms, with an 8–30% incidence (4, 5). Residual abnormal bowel, including aganglionosis and secondary neuropathological lesions in the proximal segment, are the most common causes of persistent abnormalities of bowel function (6), and the operative principles remain critical: completely removal of dysganglionic segment and advancement of normally innervated bowel to the preserved anal canal (7). Consequently, preoperative evaluation of the length of the dysganglionic segment is important in establishing the correct resection strategy, which facilitates outcomes for HSCR patients.

Major advances have taken place in the preoperative diagnosis of HSCR in recent years on the basis of anorectal manometry, radiological investigation, and rectal biopsy (8, 9). Though rectal biopsy is the gold standard for diagnosing the aganglionic length, the surgical intraoperative biopsies may suggest a negative outcome with irregular aganglionic margin. Thus, radiology still plays an important role, especially with the barium enema in demonstrating the length of the aganglionosis segment of bowel (10). Commonly, the visualization of a radiographic transition zone (TZ), described as the demarcation of the ganglionic (dilated segment) and aganglionic bowels (narrowed segment), preoperatively anticipated the range of surgical resection for an aganglionic segment. However, the fact remains that the secondary lesions of ganglia cells in the dilated segment cannot be thoroughly evaluated with TZ (11). Recently, evidence has demonstrated that the retention of barium in the colon on a 24 h delayed film also facilitates surgical planning (12).

In our center, the barium enema is considered one of the early investigations for patients with suspected HSCR, followed by a 24-h delayed film to demonstrate the length of inert bowel. In this study, we viewed the 24-h delayed film and pathological results to investigate the correlation between the retention of barium enema and the length of the dysganglionic bowel segment, providing evidence to support the guiding values of the 24-h delayed film in the surgical resection range for HSCR.

## Materials and methods

### Subjects

A retrospective study of patients (aged from 1 month to 14 years) at Tongji Hospital who underwent a preoperative 24-h delayed film of barium enema and were surgically treated from January 2015 to December 2019 was conducted. Institutional review board approval was obtained (Number: S108) from the Tongji Medical College, Huazhong University of Science and Technology.

Clinical characteristics of the children suspected of having HSCR included abdominal distention, vomiting or oral intolerance, aberrant stooling, or inability to pass meconium within 24–48 h after delivery. After clinical evaluation, anorectal manometry, contrast enema rectum, and rectal biopsy were conducted. The radiology records of these children were reviewed to identify those who had a 24-h delayed film of contrast enema performed prior to reoperation in our department. Two pediatric radiologists assessed the 24-h delayed film to determine whether there was significant barium retained, as well as the proximal level of retained barium. The records of postoperative pathological results were obtained to identify the length of abnormal bowel, and the relationship between the pathology and contrast findings was correlated.

**FIGURE 1:**
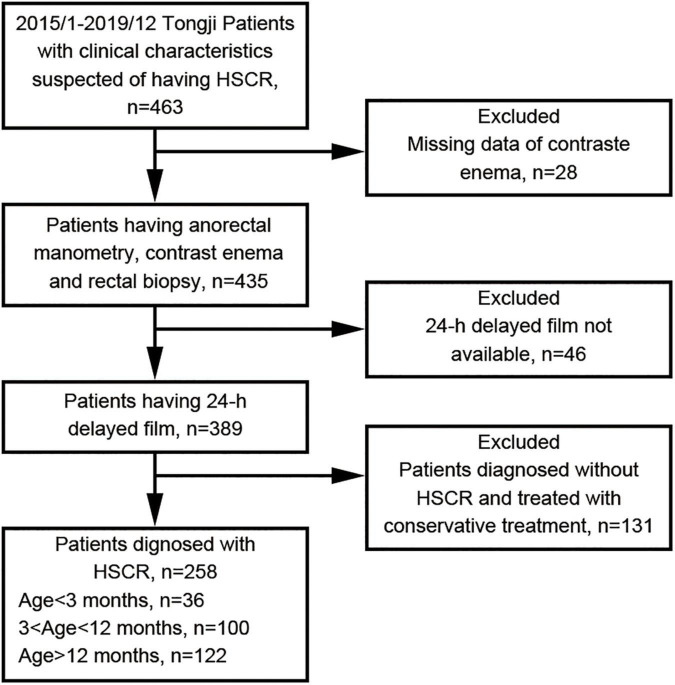
Flow chart of the study protocol.

All children younger than 14 years old with clinical suspicion for HSCR, preoperatively completed 24-h delayed film of barium enema, primary operation with pull-through procedure, pathological diagnosis approved by full thickness biopsy, no previous surgical history and no urgency were included in this study. Patients with incomplete clinical data, enterostomy or staged surgery operations, postoperative pathology results not available were excluded. Also total colon or intestinal aganglionosis was considered as exclusion criteria. Two hundred and fifty-eight children were identified as fitting these parameters. Figure 1 shows the flowchart of our study protocol.

### Radiologic and pathological evaluation

Water-diluted barium was slowly injected into the colon through a soft catheter until the shape of the total colon was clearly visible. Anteroposterior and lateral X-ray images were used to document the colon morphology, and 24-h delayed films were collected the following day. Simultaneously, the proximal location of retained barium was examined in these patients.

At our center, the surgical retention range for the bowel was 10 cm above the proximal level of retained barium. Intraoperative seromuscular biopsies of full-thickness intestinal wall was performed during surgery. Final pathological analysis of surgical resections from the distal colon, the proximal level of barium retention, and 10 cm above, respectively, was considered as confirmative diagnosis of HSCR. The ganglion cells of specimens were detected by staining for hematoxylin-eosin (HE), as described previously (13). HSCR is identified with the absence of ganglion cells in the distal colon and the presence of ganglion cells in the proximal resected bowel. A negative result was characterized as the presence of normal ganglion cells in the bowel above 10 cm of the proximal barium retention level, whereas a positive result was defined as the secondary neuropathological lesions correlated to aganglionosis or the presence of aganglionosis in the proximal barium retention level of the colon. Such secondary lesions included immature ganglia, neuronal dysplasia, hypoganglionosis, etc. Immature ganglia were identified as ganglion cells that were small and with an inconspicuous nucleolus. The neuronal dysplasia in the pathological findings was described as increased frequency of “giant” submucosal ganglia in addition to hyperganglionosis (14). Hypoganglionosis is characterized by the sparse and small myenteric ganglia, hypertrophy of muscularis mucosae, and circular muscle (15).

The association between the 24-h delayed film of barium enema and final pathological results was investigated. The sensitivity, specificity, positive predictive value (PPV), negative predictive value (NPV), Youden index and kappa value were calculated. Furthermore, the radio-histological correlation was analyzed among the different groups of HSCR patients according to the pathological outcomes, as follows: short-segment type that aganglionosis restricted within the mid-distal rectum; classical type when the aganglionic segment does not extend beyond the upper sigmoid; and long-segment type with aganglionosis extends from anal to descending colon or more proximal. Finally, children aged 3 months and younger, 3–12 months, and > 12 months were compared.

### Clinical outcomes at 1-year follow-up

Clinical outcomes of obstructive symptoms at 1-year post operation follow-up were captured from the medical records. Patients presented clinical signs of abdominal distension, bloating, vomiting, or ongoing severe constipation were identified as to have obstructive symptoms. Constipation was defined as having fewer than three bowel movements per week and/or large fecal mass in the colon at examination. Each patient with post-operative obstructive symptoms underwent a standardized diagnostic and therapeutic algorithm according to the guidelines published by Langer et al. (5) and Langer (16).

### Statistical analysis

All results were analyzed using SPSS (IBM SPSS Statistics, Armonk, NY, USA). A paired Student’s *t*-test was performed to determine the diagnostic utility of the 24-h delayed film of barium enema, compared with the final pathological results. Additionally, the correlation rate was calculated to reveal the coincidence between the 24-h delayed film of barium enema and the final pathology reports, which took into account the ages and clinical types of HSCR patients, respectively.

## Results

### Patient characteristics

A total of 463 patients were ordered owing to clinical suspicion of HSCR from January 2015 to December 2019 in our institute. Of these 463 patients, 258 (208 males and 50 females) were found to have a 24-h delayed film of barium enema and were operated with a primary pull-through procedure. The median age at diagnosis was 12 months (1–144 months). Of those, 36 (13.9%) were aged < 3 months, 100 (38.8%) were aged from 3 to 12 months, and 122 (47.3%) were aged > 12 months. The median weight of these patients was 9.0 kg (3–47 kg).

### Diagnostic outcomes

**FIGURE 2:**
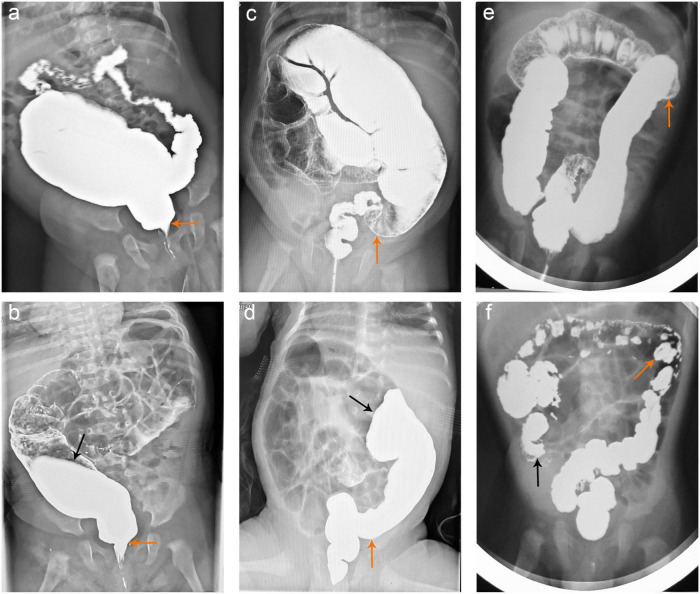
Barium enema findings in HSCR patients. **(a,b)** The barium enema demonstrated the TZ (yellow arrows) and dilated colon in a 6-month-old boy with short-segment HSCR. The 24-h delayed film revealed that the barium enema was retained up to the descending colon (black arrow). **(c,d)** The TZ characterized by barium enema was shown with yellow arrows in a 4-month-old boy with classical type HSCR, and the barium enema was retained up to the transverse colon (black arrow) in the 24-h delayed film. **(e,f)** TZ in an 8-month-old girl having long-segment HSCR was described by barium enema (yellow arrows). The 24-h delayed film indicated that the barium enema was retained up to the cecum (black arrows). HSCR, Hirschsprung’s disease; TZ, transition zone.

**FIGURE 3:**
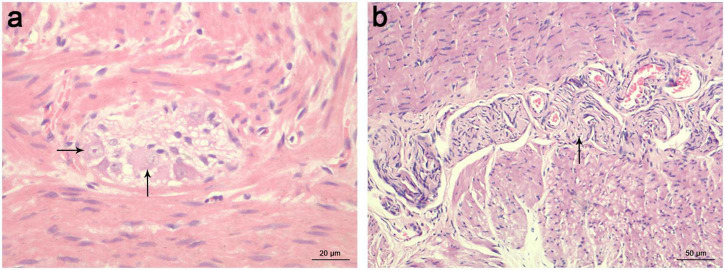
HE staining of ganglion cells in a 4-month-old boy diagnosed with HSCR. **(a)** The presence of normal ganglion cells was indicated with black arrows in the colon 10 cm above the proximal barium retention level. scale bar = 20 μm. **(b)** The absence of ganglion cells in the distal colon was replaced by hypertrophied nerve trunks, which were indicated with black arrows. scale bar = 50 μm. HE, hematoxylin-eosin; HSCR, Hirschsprung’s disease.

**FIGURE 4:**
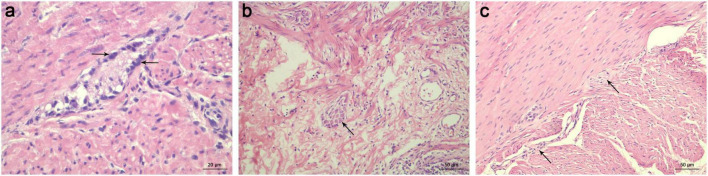
HE staining of ganglion cells in the proximal bowel with barium retention. **(a)** Immature ganglia were identified in the proximal level of barium retention. The ganglion cells appear small and have a less significant nucleus with an inconspicuous nucleolus (indicated with black arrows). scale bar = 20 μm. **(b)** Neuronal dysplasia was found in the proximal level of barium retention, described as increased frequency of “giant” submucosal ganglia (indicated with black arrows). scale bar = 50 μm. **(c)** Myenteric hypoganglionosis in the proximal level of barium retention was characterized by widely spaced small ganglia with only 1–2 ganglion cell bodies (indicated with black arrows) using HE staining. scale bar = 50 μm. HE, hematoxylin-eosin.

The 24-h delayed film of barium enema and pathological results were compared in each patient (Figure 2). Most commonly, HE staining demonstrated the absence of ganglia in the distal colon while a normal presence was 10 cm above the proximal barium retention level (Figure 3). Of the 258 patients who were suspected to have HSCR and had significant barium retained in the 24-h delayed film, 230 had positive outcomes that secondary neuropathological lesions presented in the proximal barium retention level (Figure 4), and 236 of them had negative results in the bowel 10 cm above the proximal barium retained level (Table 1). The sensitivity and specificity of the 24-h delayed film of barium enema to predict the pathological abnormal bowel was 89.1 and 91.5%, respectively. The PPV of this test was 91.3%, the NPV was 89.4%, and with a Youden index of 80.6%. Subsequently, the Kappa coefficient was calculated and yielded 0.806 (*P* < 0.001), indicated an almost perfect association between the barium findings and pathological results (Table 2).

**TABLE 1:** 24-h delayed film of barium enema and final pathological results in HSCR patients.

	Pathological result		
	Positive	Negative	Total
Proximal barium retention level	230	28	258
10 cm above the proximal barium retention	22	236	258
Total	252	264	

**TABLE 2:** Diagnostic test characteristics for predicting dysganglionic bowel segment in HSCR patients using a 24-h delayed film of barium enema.

	Value
Sensitivity	89.1%
Specificity	91.5%
Positive predictive value	91.3%
Negative predictive value	89.4%
Youden index	80.6%
Kappa value	0.806

### Correlation varies in different clinical types of Hirschsprung’s disease

To determine the guiding values of the 24-h delayed film in the surgical retention range for different clinical types of HSCR, we performed the correlation test based on the retention of barium and the pathological results of the abnormal bowel segment (Table 3). Among the 258 patients in our study, 50 of them had aganglionosis restricted to the mid-distal rectum (short-segment type). Of these 50 patients, 46 (92.0%) correlated well with the radiological discovery of the retained barium. The aganglionic segment extending up to the sigmoid colon (classical type) was identified in 186 of the cases, giving this finding a correlation rate of 93.5% (174/186). Similarly, the correlation rate between the 24-h delayed film of barium retention and pathological results in the descending colon or more proximal (long-segment type) was 72.7% (16/22). Thus, the total correlation rate was 91.5% (236/258).

**TABLE 3:** Correlation between the 24-h delayed film of barium enema and pathological results in different clinical types of HSCR.

	Pathological results			
	No evidence of HSCR	Mid-distal rectum	Sigmoid colon	Descending colon or more proximal
**24-h delayed film**				
No evidence of HSCR	0	4	12	6
Mid-distal rectum	0	46	0	0
Sigmoid colon	0	0	174	0
Descending colon or more proximal	0	0	0	16

### Correlation of the 24-h delayed film of barium enema with age

In addition, the correlation rates of the 24-h delayed film of barium enema with age were calculated. Patients younger than 3 months showed a correlation rate of 72.2% (26/36), increasing to 91.0% (91/100) and 92.6% (113/122) for patients aged 3–12 months and > 12 months, respectively. The correlation rate of female infants was 60% (6/10), while this rate (76.9%, 20/26) was higher in male patients younger than 3 months. In patients aged 3–12 months, the correlation rate in females and males was 83.3% (10/12) and 92.0% (81/88), respectively. Similarly, patients aged > 12 months showed approximately the same correlation rates in females (89.3%, 25/28) and males (93.6%, 88/94) as patients aged 3–12 months, while these were higher compared to patients younger than 3 months (Table 4). Additionally, we evaluated the sensitivity, specificity, PPV, NPV, Youden index, and Kappa value for patients younger than 3 months, which demonstrated the utility of a 24-h delayed film of barium enema in predicting the length of abnormal bowel (Tables 5, 6).

**TABLE 4:** Correlation between the 24-h delayed film of barium enema and pathological results in HSCR patients differs with age.

Age (months)	Female	Male	Total	Correlation rate (%)
<3	6/10	20/26	26/36	72.2
3–12	10/12	81/88	91/100	91.0
>12	25/28	88/94	113/122	92.6

**TABLE 5:** 24-h delayed film of barium enema and final pathological results in infants.

	Positive	Negative	Total
Proximal barium retention level	26	10	36
10 cm above the proximal barium retention	6	30	36
Total	32	40	

**TABLE 6:** Diagnostic test characteristics for predicting dysganglionic bowel segment in infants using a 24-h delayed film of barium enema.

	Value
Sensitivity	72.2%
Specificity	83.3%
Positive predictive value	81.3%
Negative predictive value	75.0%
Youden index	55.5%
Kappa value	0.556

### Clinical outcomes

To assess the clinical efficacy, medical records were reviewed and information on the obstructive symptoms at 1 year post operation was collected. A total of 44 children were excluded prior to statistical analysis of clinical outcomes due to missing postoperative data. The loss to follow-up rate at 1 year was 17.1%. Postoperative obstructive symptoms occurred in 20 cases in patients with 24-h delayed film of barium enema. The overall occurrence rate was 9.3% (20/214). Specifically, the incidence for males and females was 8.7% (15/173) and 12.2% (5/41), respectively. Among the age groups of 0–3- month-, 3–12- month-, and beyond 12-month-old patients, the rate was 0, 8.4% (7/83), and 12.9% (13/101), respectively. The numbers for short-segment, classical type, and long-segment aganglionosis HSCR cases were 7.1% (3/42), 9.2% (14/153), and 15.8% (3/19), respectively.

## Discussion

The recurrence of obstructive symptoms in HSCR patients following surgical repair highlights the importance of preoperative evaluation of the dysganglionic bowel segment. A contrast enema is commonly utilized to diagnose and guide clinical decision-making for HSCR (17). Moreover, the retention level of barium enema presented on 24-h delayed plain abdominal radiograph has been documented to be valuable in directing surgical resection range (18). However, the utility of the test that barium retained level demonstrates the dysganglionic bowel segment has never been thoroughly evaluated. Based on our findings, the 24-h delayed film of barium retention level accurately defined dysganglionic bowel segment, particularly in patients with short-segment or classical type and those older than 3 months.

The usefulness of contrast enema in the diagnosticevaluation of HSCR was first reported by Ehrenpreis (19). Numerous studies have been conducted on several characteristics of the contrast enema that are symptomatic of HSCR, including the TZ, rectosigmoid index (RSI), and irregular colorectal contractions (20). Previous studies reported debatable results regarding the accuracy of predicting abnormal bowel length using contrast enema. Some of them indicated that the length of aganglionosis on contrast enema was reliable and consistent with pathology. Green et al. showed that the level of aganglionosis in their study matched near total with pathology in 109 patients (96.2%) (21). Even though visualization of a radiographic TZ provides greater accuracy for determining the bowel resection range, 10–40% of patients continue to suffer from obstructive symptoms occurring following the surgical repair and the need for re-doing the operation (22, 23). Despite the positive outcomes of the aforementioned publications, some investigators reported cases with significant discrepancies and discordances. A discordance was observed in 25% (6/24) of SS-HSCR patients in a previous study. In all cases with discordance, it was found that contrast enema of TZ localization underestimated the exact aganglionic length (24). Similarly, Ashjaei et al. reported that radiologic TZ had always underestimated actual abnormal length in the range of 15.7–30.9 cm of bowel, which seems relatively wide (3). We believe that to avoid more complications, a highly accurate strategy for perioperative evaluation of dysganglionic bowel segment is required.

Previous studies have demonstrated that the neuropathological features immediately proximal to the aganglionic segment seem to correlate with postoperative dysmotility. Estevão-Costa et al. reported that 46% of HSCR cases have other dysganglionoses in the proximal ganglionic colon by using appropriate histochemical methods, which has been associated with persistent postoperative constipation and obstruction (25). They recommended that histochemical mapping of the entire colon be performed before definitive pull-through to prevent surgical failure in HSCR. Similarly, their following study emphasized histochemical characterization of the proximal colon with no radical resection of the neuronal dysplasia segment seems to be an effective and safe approach to minimize the prevalence of postoperative enterocolitis (26). Furthermore, a study performed by Carvalho et al. indicated that laparoscopic-assisted mapping of the entire colon may contribute to improving the outcome of intestinal dysganglionosis by better characterization of the disease (27). In recent years, our center has emphasized the 24-h delayed film of barium enema as a critical component of HSCR preoperative management due to its clinical convenience, effectiveness, and cost savings in preoperatively identifying secondary lesions of ganglia cells in dilated segments. Principally, the surgical retention range for the bowel is 10 cm above the proximal level of retained barium on the 24-h delayed film in our institution.

In our study, the correlation rate between the barium retained level and the pathological outcomes of dysganglionic bowel segment was up to 91.5%, followed by a sensitivity and specificity of 89.1 and 91.5%, respectively. The occurrence rate of obstructive symptoms was 9.3% in the present study, which was consistent with previous reported 8–30% incidence in children early or years after their initial pull-through surgery. Thus, we inferred that delayed film of barium enema might not perform well in reducing long-term postoperative complications, as there were several factors associated with postoperative obstruction, including mechanical obstruction, persistent or acquired aganglionosis, internal sphincter achalasia, and functional megacolon. This study validated the diagnostic role of 24-h delayed film in predicting dysganglionic bowel length pre-operation.

Our preliminary results suggest the usefulness of 24-h delayed film in predicting inert bowel and guiding surgical operations. Additionally, there is a significantly higher correlation with aganglionosis restricted to the rectum (short-segment type, 92.0%) and the sigmoid colon (classical type, 93.5%) compared to those in the descending colon or more proximal (long-segment type, 72.7%). This may be due to the fact that aganglionosis is more widespread in patients with long-segment HSCR. However, even though the correlation rate is relatively lower in the descending colon, it is still significant when compared to our previous study, which found a correlation rate of 44.4% between the TZ on barium enema and the pathological TZ in the descending colon (28). Generally, the 24-h delayed film of barium retained level is more accurate than TZ on barium enema to predict dysganglionic bowel segment in the descending colon or more proximal. Thus, we propose that the length of bowel resection should be extended and validated by intraoperative frozen sections in patients with long-segment HSCR.

Compared to older children with HSCR, infants have a shorter course of disease, making a radiological study less explicit and visible. According to the findings of the current study, patients younger than 3 months had lower sensitivity and specificity, and the correlation rate between 24-h delayed film of barium retention and pathological results decreased as well, particularly in female infants. A probable explanation is that a dilated colon with secondary neuropathological lesions requires time to develop before it can be reflected in a contrast enema, and thus may not be noticeable in the early months. In addition, Proctor et al. have reported similar findings. Of the eight patients who had a TZ that was significantly different from the level of aganglionosis detected intraoperatively, the majority (7 of 8, 88%) of discordant studies occurred in infants less than 1 month of age (22). Despite this, positive contrast enema significantly increases the probability of infants receiving a diagnosis of HSCR from 13% with clinical suspicion alone to 82% (29). Putnam et al. reported a diagnostic accuracy of 70% with any two of the findings in RSI, TZ, and the “sawtooth” pattern on contrast enema, which increased to 90% with all three findings being present in neonates. Lastly, we believe that the accuracy of diagnosis for HSCR in infants will be improved when a 24-h delayed film of barium enema is combined.

There are some limitations in our study. As a single-center, retrospective study, the results are subject to temporal confounders and other methodological flaws. Lastly, further large multicenter studies and randomized clinical trials will also be designed to corroborate our results.

## Conclusion

Our review of the 24-h delayed film of barium enema performed for patients suspected of having HSCR demonstrated that the barium retention level remains crucial in predicting dysganglionic bowel segment, which contributes to the decision-making for surgical physicians. Even though the correlation rate varies with the age and clinical type of HSCR patients, the level of retained barium is a useful predictor for actual disease involvement, and histopathology is still necessary for determining the exact dysganglionic bowel segment in HSCR patients.

## Data availability statement

The raw data supporting the conclusions of this article will be made available by the authors, without undue reservation.

## Ethics statement

The studies involving human participants were reviewed and approved by the Tongji Medical College, Huazhong University of Science and Technology. Written informed consent to participate in this study was provided by the participants’ legal guardian/next of kin. Written informed consent was obtained from the minor(s)’ legal guardian/next of kin for the publication of any potentially identifiable images or data included in this article.

## Author contributions

BZ was responsible for extracting and analyzing data, screening potentially eligible patients, interpreting results, and drafting the manuscript. DW was responsible for data analysis and extraction, interpreting the results, and providing feedback on the report. KC, YN, and CJ contributed to data extraction and provided feedback on this report. TZ and JF were responsible for designing the protocol, conducting the research, and revising the manuscript. JF contributed to funding acquisition and the whole project supervision as well. All authors contributed to the article and approved the submitted version.
